# Patterns of Medication Dispensation for Multiple Comorbidities among Older Adults in Australia

**DOI:** 10.3390/pharmacy6040134

**Published:** 2018-12-17

**Authors:** Richard Ofori-Asenso, Jenni Ilomaki, Andrea J. Curtis, Ella Zomer, Sophia Zoungas, Danny Liew

**Affiliations:** 1 Centre of Cardiovascular Research and Education in Therapeutics, Department of Epidemiology and Preventive Medicine, Monash University, Melbourne, VIC 3004, Australia; richard.ofori-asenso@monash.edu (R.O.-A.); ella.zomer@monash.edu (E.Z.); 2 Epidemiological Modelling Unit, Department of Epidemiology and Preventive Medicine, Monash University, Melbourne, VIC 3004, Australia; 3 Centre for Medicine Use and Safety, Faculty of Pharmacy and Pharmaceutical Sciences, Monash University, Melbourne, VIC 3052, Australia; jenni.ilomaki@monash.edu; 4 Department of Epidemiology and Preventive Medicine, Monash University, Melbourne, VIC 3004, Australia; 5 Division of Metabolism, Genomics and Ageing, Department of Epidemiology and Preventive Medicine, Monash University, Melbourne, VIC 3004, Australia; andrea.curtis@monash.edu (A.J.C.); sophia.zoungas@monash.edu (S.Z.)

**Keywords:** multimorbidity, older adults, disease clusters, comorbidity, medications, Australia

## Abstract

**Background:** The increasing burden of chronic (medical) conditions (CCs) is a major issue for healthcare systems across the world. We aimed to examine the changes in the rate of medication dispensation for multiple CCs among Australians aged ≥65 years. **Methods:** A repeated cross-sectional study was performed using the 2013–2016 Pharmaceutical Benefits Scheme (PBS) data on reimbursed prescriptions for a 10% random sample of the Australian population. Twenty-two CCs were identified via the RxRisk-V tool. The yearly changes in the proportion of older adults dispensed medications for ≥2 CCs were determined through Poisson regression modelling using 2013 as the reference year. The occurrence of CC dyads and triads for which medications were dispensed within a 180-day window were characterised, and the observed and expected rate of medication dispensation for each CC dyad or triad were calculated to identify the top 15 combinations. **Results:** The proportion of older adults dispensed medications for ≥2 CCs remained stable from 2013 to 2016, at >79% in each year. The proportion who were dispensed medications for multiple CCs increased with age. No gender differences in the dispensation of medications for multiple CCs were observed. Over 60% had medications dispensed for ≥3 CCs. The most frequent CC dyad and triad for which medications were dispensed were dyslipidaemia + hypertension (38.6%) and dyslipidaemia + gastroesophageal reflux disease + hypertension (18.7%), respectively. For the majority of CC dyads and all triads examined, the observed rate of medication dispensation exceeded that expected by chance. **Conclusions:** A high proportion of older Australians are dispensed medications for multiple CCs, suggestive of multimorbidity. The results reiterate the need for increased research into understanding the causal mechanisms of multimorbidity to inform the design of cost-effective interventions.

## 1. Background

Like most developed countries, Australia is experiencing significant population ageing. At the turn of the 20th century, just 1 in 25 Australians was aged ≥65 years; today, the number stands at 1 in 6, and it is projected to reach 1 in 4 by 2050 [1].

Older age is associated with an increased risk of chronic morbidities. Thus, multimorbidity—defined as the presence of two or more chronic (medical) conditions (CCs) in an individual measured at the same time without defining an index disease [2]—is common among older adults. A recent systematic review based on data mainly from North America and Europe found that among older adults, more than 66% had multimorbidity [3], and that this poses a major challenge for healthcare systems across the world.

Understanding the burden of individual CCs (and multimorbidity) and the consequent healthcare utilisation patterns among the older population is important for informing preventive strategies. Similarly, such information is critical to healthcare planning and policy formulation. Within Australia, the biennial National Health Survey also reports a high burden of chronic ailments among Australians [4,5], although such studies are typically susceptible to selection bias and data misclassification due to self-reporting. Data from national disease registries that include other clinical diagnoses are equally useful to provide insights into both disease burden (incidence, prevalence and mortality) as well as the burden of treatment (i.e., the workload of healthcare and its effect on patient functioning and wellbeing) [6]. However, for many diseases, registries do not exist, are not well developed, do not adequately capture comorbid conditions, or lack adequate coverage of the Australian population [7].

Recently, administrative information, such as that from medication claims data, has been proposed to be useful in identifying persons with CCs [8,9]. Pharmacy-based medication acquisition data are often readily available and have high coverage. Studies conducted in several countries, including Switzerland [10,11], Italy [9,12,13], Ireland [14] and the United States [15,16], suggest that medication claims data may be useful for characterising the burden of individual CCs and multimorbidity.

There have been earlier studies which have used pharmacy claims data to estimate the prevalence of individual CCs among selected Australian populations [17,18,19]. Limited studies have, however, examined the trends in the dispensation of medications for multiple CCs. In addition, there is an increasing call for research into characterising the patterns of CC combinations or clusters for which medications are being dispensed [20]. In particular, the identification of the most common CC combinations for which people are being dispensed medications provides insight into the patterns of CC clustering, which in turn will inform preventive approaches [21]. Furthermore, such data could help clinicians whose specialty focuses on single diseases to know what other comorbidities are likely to be present among their patient populations so as to coordinate their care with other clinicians.

In the current study we sought to (i) assess the changes in the proportion of older adults who are dispensed medications for multiple CCs over the period 2013–2016; and (ii) characterise the most frequently occurring CC dyads and triads for which medications are being dispensed.

## 2. Method

### 2.1. Study Design and Data Source

A repeated cross-sectional study was conducted using Pharmaceutical Benefits Scheme (PBS) claims data for the period 2013–2016 in a 10% random sample of the Australian population [22]. The PBS subsidises the costs of prescription medications for all citizens and permanent residents in Australia. All PBS claims submitted for payment of a government subsidy are processed by the Australian Department of Human Services for monitoring, evaluation and planning purposes [22]. Data in the PBS datasets are drawn from administering pharmacies or hospitals [22]. The information captured in the PBS datasets includes medication item codes, the names and strengths of dispensed medications and demographic information (year of birth, sex and year of death if applicable). Also included are prescriber identification codes, the date of dispensing, the quantity of medication supplied, the state or territory of the pharmacy, and the co-payment and beneficiary status. The data does not contain any information on clinical diagnosis nor laboratory test results. Data used in the study were curated and supplied by the Australian Department of Human Services. 

### 2.2. Study Population

The study population included all persons aged 65 years and over who were included in the 10% random sample of the PBS data between 1 January 2013 and 31 December 2016. 

### 2.3. Study Outcomes

We estimated the proportions of older adults dispensed medications for a pre-determined set of 22 CCs: hypertension, gout, glaucoma, congestive heart failure, dementia, Parkinson’s disease, reactive airway disease, gastroesophageal reflux disease (GORD), depression, anxiety, arrhythmias, epilepsy, angina, dyslipidaemia, steroid responsive conditions, hyperthyroidism, malignancies, diabetes, psychotic illness, osteoporosis, inflammatory disorders (pain/inflammation) and pain. The CCs were selected for one or more of the following reasons: (i) they were identified in prior cohort studies and national surveys to be highly prevalent [4,5]; (ii) they are associated with high healthcare utilisation [23,24]; (iii) they contribute to significant disability or deaths among older Australians [4,17,21,25,26]; and (iv) medication mapping for these comorbidities has been validated within the Australian context [27,28]. Previous research has indicated that the inclusion of any 12 or more prevalent CCs is sufficient to estimate the burden of disease (i.e., multimorbidity) among any population adequately [29]. The RxRisk-V, a co-morbidity prescription-based measure that utilises medication histories to determine the presence of conditions, was employed in this study [27,28]. The specific codes used in identifying individual CCs are presented in Supplemental Table S1. The proportion of older adults dispensed medications for individual and multiple CCs was estimated annually from 2013 to 2016.

We used a 180-day window to estimate the proportion of older people concurrently dispensed medications for multiple CCs; that is, for a person to have medication dispensed for CC dyad XY, they must have had medication dispensed for X and Y within a 180-day period. Because people could discontinue medication at any time [30], we chose the 180-day period to ensure that dispensed medications for different CCs were likely to have been used around the same period. Thus, data for July–December 2016 were used to determine the proportion of people with each CC who were dispensed medications for other CCs. The occurrence of CC dyads and triads for which older adults were being dispensed medications were examined by clustering the CC per individual and estimating the prevalence for each combination. We focused on the 15 most prevalent CC dyads and triads for which medications were dispensed. The expected prevalence was calculated by multiplying the proportion of people dispensed medication for the single CC within the dyads or triads by each other (e.g., for CC dyad XY, the expected prevalence of people dispensed medication was calculated as the proportion of people dispensed medication for condition X multiplied by the proportion of people dispensed medication for condition Y) [20]. This assumes that individual CCs are independent of one another. In a sensitivity analyses, we varied the window to 365 days using the records for January–December 2016 to ascertain if the observed patterns of medication dispensation for multiple CCs would change. 

### 2.4. Statistical Analyses

Descriptive population characteristics in respective years were summarised using frequency tables for categorical variables and summary statistics (mean with standard deviation (SD)) for continuous variables. The proportion of older adults dispensed medications for individual CCs and their aggregation (0, 1, 2, 3, 4, 5, 6, 7, 8, 9, ≥10), multimorbidity (≥2 CCs) and CC dyads and triads are presented. For the yearly analyses, a person must have been dispensed ≥1 script for any of the medications indicated for that CC within the calendar year before being assigned as having that CC. The proportion dispensed medication for multiple CCs was determined for the total population and stratified by age-group (65–74, 75–84 and ≥85 years) and sex. The relative changes in the proportion of older adults dispensed medication for individual and multiple CCs during 2013–2016 were assessed via Poisson regression modelling using 2013 as the reference year, with adjustments for age and sex. Using the data from July–December 2016, for each of the 22 CCs, the proportions of people dispensed medication for 0, 1, 2, 3 and 4 or more other CCs were calculated. The observed and expected proportion of the older adults dispensed medications for each CC dyad or triad was compared via a two-sample test of proportion [31]. All analyses were performed using Stata software (Stata/IC v14.1; StataCorp, College Station, TX, USA). The study received approval from the Monash University Human Research Ethics Committee.

## 3. Results

### 3.1. Sample Characteristics

The characteristics of the older adults in each year are presented in Table 1. The number of older adults in the 10% PBS sample increased from 315,074 in 2013 to 351,471 in 2016. There were more women than men in each year. The mean age remained stable across the study timeframe. 

### 3.2. Patterns of Medication Dispensation for Individual CCs from 2013 to 2016

Of the 22 CCs examined, the CC for which medications were most likely to be dispensed was hypertension (> 65%) and the least was hyperthyroidism (<1%) (Table 2). The Poisson regression models showed a slight increase in the likelihood of an older adult being dispensed medication for diabetes, dementia, malignancy and steroid responsive conditions (Supplementary Table S2). On the other hand, there was a slight decrease in the likelihood of an older adult being dispensed medications for anxiety, angina and epilepsy, whiles the likelihood of being dispensed medication for the remaining CCs remained stable.

### 3.3. Dispensation of Medications for Multiple CCs

The mean number of CCs for which medications were dispensed among the sample population was stable at 3.4 ± 2.1 in all years. In each year, fewer than 7% of persons were dispensed medications for none of the studied CCs, while around 15% were dispensed medications for one CC. The proportion of older adults dispensed medications for ≥2 of the studied CCs ranged from 79.5% in 2016 to 79.8% in 2013. Compared to 2013, older Australians in 2014–2016 were no more likely to be dispensed medications for 2 or more CCs. In the 2013–2016 period, there was no significant gender differences in the likelihood of being prescribed medications for multiple CCs (age-adjusted rate ratio (aRR) of men compared to women, 0.99, 95% confidence interval (CI) 0.99–1.00). However, compared to those aged 65-74 years, Australians aged 75–84 (sex-adjusted rate ratio (sRR) 1.16, 95% CI 1.15–1.16) and ≥85 years (sRR 1.18, 95% CI 1.17–1.19) were more likely to be dispensed medications for ≥2 CCs. In each year between 2013 and 2016, more than 60% of older Australians were dispensed medications for three or more of the studied CCs (Table 3), and over a quarter were dispensed medications for five or more CCs. 

### 3.4. Patterns of Medication Dispensation for Multiple CCs

**Figure 1:**
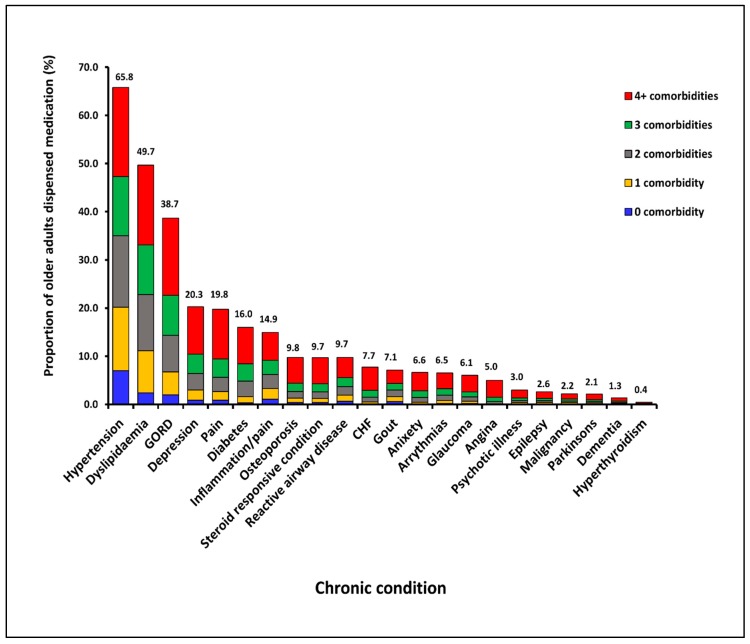
Prevalence of individual CCs with the number of co-morbidities among older Australians captured in the PBS datasets in July–December 2016.

Figure 1 shows the proportion dispensed medication for each CC and also displays the proportions of people dispensed medication for each CC who were dispensed medication for other CCs using data for the period July–December, 2016. At least 89.4% (range across individual CCs 89.4–99.1%) of people who were dispensed medication for one of the CCs also had medication dispensed for at least one other CC within the 180-day period. Furthermore, approximately three quarters of people who were dispensed medications for one of the CCs also had medications dispensed for at least two other CCs.

We also observed 231 unique CC dyads for which medications were dispensed among the older adults. Table 4 shows the observed and expected prevalence of pairs of CCs with medications dispensed and the O/E ratios of the top 15 dyads ranked by observed prevalence. The most prevalent CC dyads for which medications were dispensed were hypertension + dyslipidaemia (38.6%), followed by GORD + hypertension (27.6%) and dyslipidaemia + GORD (22.1%). Six of the 15 most prevalent dyads involved hypertension, dyslipidaemia or GORD. The medication dispensation rate for 13 out of the 15 CC dyads (87%) occurred more frequently than would have been expected (*p*-value for difference <0.05).

A total of 1539 unique CC triads for which medications were dispensed were observed. Table 5 displays the observed and expected prevalence of triads of CCs with medications dispensed and the O/E ratios of the most commonly occurring 15 triads. The most frequently occurring triads for which medications were dispensed were dyslipidaemia + GORD + hypertension (18.7%), diabetes + dyslipidaemia + hypertension (10.8%) and dyslipidaemia + pain + hypertension (10.8%). Dyslipidaemia occurred in nine out of the top 15 triads, whereas hypertension occurred in ten and depression in five. The rate of medication dispensation for all the 15 CC triads occurred more frequently than expected (*p*-value for difference <0.05). Varying the assessment window to 365 days did not change the patterns of medications dispensed for CC dyads and triads. 

## 4. Discussion

We investigated the occurrence of medication dispensation for one or more of 22 CCs among older Australians over the period 2013–2016 using a nationally representative medication claims dataset. We found that 79% of older adults were dispensed medications for ≥2 CCs, and this remained stable over the four-year period. 

Our results highlight that over three-quarters of older adults may have multimorbidity. This rate is higher than those reported in recent National Health Surveys [32], as well as the prevalence noted in prior cross-sectional studies [21,33]. In these studies, which relied on self-reported data, 60–65% of older adults self-reported having 2 or more CCs. However, respondents may be more likely to report only symptomatic illness, and older age has been demonstrated to be associated with a decreased accuracy of self-reported health status, regardless of cognitive status [2]. Additionally, some of the self-reported studies included fewer CCs (for example, considering all cardiovascular diseases together), and multimorbidity prevalence has been demonstrated to be sensitive to the number of CCs included in the assessment [29]. Overall, our study results concur with those of earlier studies, providing evidence that multimorbidity is a significant issue among older Australians [21,34].

We found that the dispensation of medications for certain CC pairs (e.g. dyslipidaemia + hypertension) occurred more frequently than the rate of medication dispensation for individual CCs such as glaucoma, gout and epilepsy. Also, we noted that among persons with a dispensed medication for one of the studied CCs, more than 3 in 4 had medications dispensed for at least two of the other 22 CCs. This high proportion of individuals with medication dispensed for concurrent CCs reiterates the need to refocus the design of health services, which tend to be single-disease focused, as well as in guideline development [35,36].

For the majority of CC dyads and all triads, the rate of medication dispensation exceeded that expected by chance. This finding supports previous research which has demonstrated that certain CCs such as hypertension and depression are frequently clustered [37]. Another possible explanation for the higher observation of the dispensation of medications for CC dyads and triads may be that once an individual has been diagnosed with a first or second CC, frequent engagement with clinicians for management and monitoring may provide further opportunity for the diagnosis of other CCs [35].

The high proportion of older adults dispensed medications for multiple CCs imposes a significant financial burden on the Australian health care system. The total healthcare expenditure in Australia nearly doubled from AUD $59.1 billion in 2005–2006 to AUD $114.6 billion in 2015–2016 [38]. The older population have been significant contributors to the rising health expenditure due to the higher burden of CCs. For example, in 2008–2009, health expenditure in adult Australians aged 85+ was almost 20 times higher compared to that for persons aged 5–14 years [39]. The overall financial impact of multimorbidity on the Australian healthcare system and economy has not been thoroughly quantified. However, previous research found that older Australians with multimorbidity spend around 2.5–5.7 times as much on their health than those with no diagnosed CC; each additional CC also increased the likelihood of a person facing severe financial burden due to healthcare costs by 46% [33].

It has been argued that government commitment and funding for disease prevention is insufficient [40]. In particular, more attention on primary prevention (in particular, focusing on eliminating known risk factors, e.g., poor dietary practices in the case of diabetes) in the older cohort to delay the onset of CCs and increase disability-free survival, may ultimately reduce the cost burden on the Australian healthcare system. Such efforts must, however, look beyond individual CCs and be directed towards reducing overall multimorbidity and improving quality of life [41].

Our study has some strengths. We utilised a large, nationally representative medication claim data set to describe the patterns of medication dispensation for CCs among older Australians. Use of claims-based data has the advantage of deriving results that reflect the burden of disease among those actively utilising health services or those who may have frequent need for such services [29,42]. CCs were also identified using the Rx-Risk-V tool, which has been widely used and validated in Australia and other countries [17,18,27]. The use of claims-based data allowed the inclusion of a large sample of older adults that is unlikely to be achieved via direct patient interviews. 

The main limitation of our study was that CCs were identified solely on the basis of medication records. While this has the advantage of identifying persons who may have reached a threshold of impairment or risk requiring therapy, we may have missed persons who are otherwise not on medications for specific CCs. Also, for some CCs, prescribing coverage of drug therapy is low and thus a prevalence based on medication use rate may not be reflective of the actual disease burden. An example is the use of anticholinesterase agents for dementia. In our analysis, less than 2% of older Australians were dispensed dementia medication, although the prevalence of dementia among Australians aged 65 years and over has been reported to be around 10% [43]. In a similar US study, the prevalence of dementia was estimated to be <1% using claims-based data compared to about 5% when prevalence was estimated from other data sources [44]. Moreover, our analyses are based on only older adults who were dispensed PBS medication. This is likely to lead to a slight over-estimation of multimorbidity, as persons not dispensed medications are likely to have been excluded. We posit, however, that for the population under-consideration, this may be of a minimal impact. Finally, our analyses did not consider individual CC severity or associated healthcare utilisation.

## 5. Conclusions

The dispensation of medications for multiple CCs is highly prevalent among older Australians, although the rate remained stable during the period 2013–2016. The proportion of older adults dispensed medications for certain CC combinations are more prevalent than certain individual conditions that attract a lot of attention. Increased preventive measures are required to reduce the onset of CCs and multimorbidity and avert/decrease the significant health and economic burden. Further research aimed at also assessing the causal mechanisms of multimorbidity may help to design cost-effective interventions.

## Figures and Tables

**Table 1:** Descriptive characteristics of older adults in the Pharmaceutical Benefits Scheme (PBS) datasets from 2013 to 2016.

Year	Total No. of Persons	Mean Age (S.D), Years	Gender Distribution					
			Men (n, %)			Women (n, %)		
			65–74	75–84	≥85	65–74	75–84	≥85
**2013**	315,074	72.7 (7.5)	84,356 (26.8)	46,514 (14.8)	13,877 (4.4)	89,572 (28.4)	55,104 (17.5)	25,651 (8.1)
**2014**	327,433	73.7 (7.5)	87,557 (26.7)	47,982 (14.7)	15,342 (4.7)	93,467 (28.5)	56,286 (17.2)	26,799 (8.2)
**2015**	340,153	73.8 (7.5)	91,060 (26.8)	49,584 (14.6)	16,586 (4.9)	97,434 (28.6)	57,510 (16.9)	27,979 (8.2)
**2016**	351,471	73.8 (7.5)	93,880 (26.7)	51,193 (14.6)	17,640 (5.0)	100,808 (28.7)	59,188 (16.8)	28,762 (8.2)

**Table 2:** Proportion of older adults dispensed medications for chronic conditions from 2013 to 2016.

Chronic Condition	2013	2014	2015	2016
Hypertension	66.5	66.2	65.8	65.7
Dyslipidaemia	50.9	50.3	50.0	50.0
GORD	41.0	41.4	41.3	41.4
Pain	25.7	26.5	26.9	26.6
Depression	21.4	21.4	21.6	21.8
Inflammation/pain	20.8	20.5	20.2	20.1
Diabetes	15.7	15.9	16.1	16.3
Steroid responsive conditions	12.3	12.7	13.1	13.3
Reactive airway disease	11.9	12.1	12.1	12.0
Osteoporosis	10.5	10.4	10.5	10.8
CHF	9.0	8.9	8.8	8.8
Anxiety	8.7	8.4	8.0	7.9
Glaucoma	7.6	7.5	7.4	7.3
Gout	7.4	7.4	7.5	7.5
Angina	7.3	6.9	6.5	6.3
Arrythmias	6.6	6.6	6.8	7.1
Psychotic illness	3.7	3.6	3.6	3.5
Epilepsy	3.1	3.0	3.0	2.9
Malignancy	2.3	2.4	2.5	2.6
Parkinson’s disease	2.2	2.3	2.3	2.3
Dementia	1.3	1.4	1.4	1.5
Hyperthyroidism	0.5	0.5	0.5	0.5

CHF = congestive heart failure; COPD = Chronic obstructive pulmonary disease; GORD = gastroesophageal reflux disease.

**Table 3:** Distribution of number of chronic conditions with medication dispensed within each age and sex group from 2013 to 2016.

No. of CCs	Percentage of Older Adults Dispensed Medications for No. of CCs in Respective Years																							
	2013						2014						2015						2016					
	All	M	W	65–74	75–84	85+	All	M	W	65–74	75–84	85+	All	M	W	65–74	75–84	85+	All	M	W	65–74	75–84	85+
0	5.7	5.9	5.6	7.6	3.4	3.3	5.8	6.0	5.7	7.8	3.5	3.2	5.9	5.9	5.8	7.9	3.4	3.1	5.8	5.9	5.7	7.8	3.4	3.2
1	14.6	15.2	14.0	17.9	10.7	9.6	14.7	15.3	14.2	18.1	10.8	9.3	14.7	15.2	14.3	18.1	11.0	9.3	14.7	15.2	14.2	18.1	10.9	9.3
2	18.5	19.1	18.0	20.5	16.5	14.8	18.2	18.9	17.7	20.2	16.3	14.4	18.2	18.7	17.8	20.2	16.4	14.3	18.3	18.8	17.7	20.2	16.4	14.4
3	18.6	19.0	18.2	18.8	18.6	17.7	18.5	18.9	18.2	18.6	18.7	17.8	18.5	18.9	18.1	18.4	18.8	17.8	18.5	19.0	18.2	18.6	18.8	17.9
4	15.5	15.4	15.6	14.1	17.2	17.4	15.5	15.5	15.6	14.1	17.0	17.8	15.5	15.5	15.5	14.1	17.0	17.7	15.5	15.5	15.6	14.1	17.1	17.7
5	11.3	10.8	11.7	9.4	13.3	14.4	11.3	10.9	11.8	9.5	13.3	14.4	11.4	11.0	11.7	9.5	13.2	14.8	11.4	11.0	11.7	9.5	13.3	14.6
6	7.4	6.9	7.8	5.7	9.2	10.4	7.4	7.0	7.8	5.6	9.3	10.5	7.4	7.0	7.8	5.8	9.2	10.2	7.4	7.0	7.8	5.7	9.2	10.4
7	4.4	4.0	4.7	3.2	5.5	6.4	4.4	4.0	4.7	3.2	5.6	6.6	4.4	4.0	4.7	3.2	5.6	6.7	4.4	4.0	4.7	3.2	5.6	6.5
8	2.3	2.1	2.5	1.6	3.1	3.5	2.3	2.0	2.5	1.6	3.0	3.4	2.3	2.1	2.5	1.6	3.0	3.5	2.3	2.1	2.5	1.6	3.0	3.4
9	1.1	1.0	1.2	0.7	1.6	1.6	1.1	1.0	1.2	0.8	1.5	1.5	1.1	1.0	1.2	0.8	1.4	1.7	1.1	1.0	1.2	0.8	1.5	1.6
≥10	0.7	0.7	0.7	0.5	1.0	1.0	0.7	0.7	0.7	0.5	1.0	1.0	0.7	0.7	0.7	0.5	1.0	0.9	0.7	0.7	0.7	0.5	1.0	1.0
Total	100	100	100	100	100	100	100	100	100	100	100	100	100	100	100	100	100	100	5.8	5.9	5.7	7.8	3.4	3.2
Mean no. of CCs (S.D)	3.4 (2.1)	3.3 (2.1)	3.4 (2.1)	3.0 (2.0)	3.8 (2.1)	3.9 (2.2)	3.4 (2.1)	3.3 (2.1)	3.4 (2.1)	3.0 (2.0)	3.8 (2.1)	4.0 (2.1)	3.4 (2.1)	3.3 (2.1)	3.4 (2.1)	3.0 (2.0)	3.8 (2.1)	4.0 (2.1)	3.4 (2.1)	3.3 (2.1)	3.0 (2.0)	3.1 (2.1)	3.8 (2.1)	4.0 (2.1)
% ≥2 CC	79.8	79.0	80.4	74.5	85.9	87.1	79.5	78.8	80.1	74.1	85.7	87.4	79.4	78.9	79.9	74.0	85.6	87.6	79.5	78.9	80.1	74.1	85.7	87.6

CC= chronic conditions; M= men; W= women; s.d = standard deviation.

**Table 4:** Observed and expected rate of medication dispensation for CC dyads among older Australians (July–December 2016).

Ranking *	CC Dyad	Prevalence (%)		O/E Ratio	*p*-Value ^†^
		Expected (E)	Observed (O)		
1.	Dyslipidaemia + hypertension	29.8	36.0	1.21	<0.001
2.	Gastric acid disorder + hypertension	24.7	27.1	1.10	<0.001
3.	Dyslipidaemia + gastric acid disorder	20.8	23.9	1.15	<0.001
4.	Pain + hypertension	15.9	16.7	1.05	<0.001
5.	Gastric acid disorder + pain	11.0	14.8	1.35	<0.001
6.	Dyslipidaemia + pain	13.3	13.8	1.04	<0.001
7.	Depression + hypertension	12.9	13.5	1.05	<0.001
8.	Diabetes + dyslipidaemia	8.2	12.8	1.56	<0.001
9.	Diabetes + hypertension	9.7	12.5	1.29	<0.001
10.	Depression + gastric acid disorder	9.0	12.4	1.38	<0.001
11.	Depression + dyslipidaemia	10.9	11.9	1.09	<0.001
12.	Inflammatory disorder + hypertension	12.0	11.5	0.96	<0.001
13.	Dyslipidaemia + Inflammation/pain	10.1	9.9	0.98	0.0059
14.	Gastric acid disorder + inflammation/pain	8.3	9.9	1.19	<0.001
15.	Depression + pain	5.8	8.9	1.53	<0.001

* Results by order of observed frequency; ^†^ *p*-values between the observed and expected prevalence were obtained using a two-sample test of proportion.

**Table 5:** Observed and expected rate of medication dispensation for CC triads among older Australians (July–December 2016).

Ranking *	CC Triad	Prevalence (%)		O/E Ratio	*p*-Value ^†^
		Expected (E)	Observed (O)		
1.	Dyslipidaemia + GORD + hypertension	12.7	18.7	1.47	<0.001
2.	Diabetes + dyslipidaemia + hypertension	5.2	10.8	2.08	<0.001
3.	Dyslipidaemia + pain + hypertension	6.5	10.8	1.66	<0.001
4.	GORD + pain + hypertension	5.0	10.6	2.12	<0.001
5.	Dyslipidaemia + GORD + pain	3.8	9.1	2.39	<0.001
6.	Depression + dyslipidaemia + hypertension	6.6	9.0	1.36	<0.001
7.	Depression + GORD + hypertension	5.2	8.7	1.67	<0.001
8.	Depression + dyslipidaemia + GORD	3.9	7.8	2.00	<0.001
9.	Dyslipidaemia + inflammation + hypertension	4.9	7.3	1.49	<0.001
10.	Diabetes + dyslipidaemia + GORD	3.1	6.7	2.16	<0.001
11.	GORD + inflammation + hypertension	3.8	6.6	1.74	<0.001
12.	Diabetes + GORD + hypertension	4.1	6.5	1.59	<0.001
13.	Depression + GORD + pain	1.6	6.3	3.94	<0.001
14.	Depression + pain + hypertension	2.6	6.1	2.35	<0.001
15.	Dyslipidaemia + GORD + inflammation/pain	2.9	5.9	2.03	<0.001

* Results by order of observed frequency; GORD = gastroesophageal reflux disease; ^†^ *p*-values between the observed and expected prevalence were obtained using a two-sample test of proportion.

## Supplementary Materials

The following are available online at http://www.mdpi.com/2226-4787/6/4/134/s1, Table S1: RxRisk-V categories, Table S2: Results of poisson regression models examining the likelihood of an older adult being dispensed medication for individual chronic conditions for 2014–2016 compared to 2013*.
