# Supplementary material for: Patterns of Medication Dispensation for Multiple Comorbidities among Older Adults in Australia
Source: Pharmacy (Basel). 2018 Dec 17;6(4):134. doi: 10.3390/pharmacy6040134 (PMC6306834; doi:10.3390/pharmacy6040134)
Supplement: Supplementary file 1 [file pharmacy-06-00134-s001.pdf]

Table S1. RxRisk-V categories.

Arrhythmias	C01AA05, C01BA01–C01BD01, C07AA07
CHF	(C03CA01–C03CC01 AND (C09AA01–C09AA16 OR, C09CA01–C09CX99)), C03DA04, C07AB07, C07AG02, C07AB12, C09DX04, C07AB02_2**
Dementia	N06DA02–N06DA04, N06DX01
Depression	N06AA01–N06AG02, N06AX03–N06AX11, N06AX13–N06AX26
Diabetes	A10AA01–A10BX08
Gastroesophageal reflux disease	A02BA01–A02BX05
Glaucoma	S01EA01–S01EB03, S01EC03–S01EX02
Gout	M04AA01–M04AC01
HTN	C03AA01–C03BA11, C03BB04, C03DA01–C03DA03, C03EA01–C03EA14, C09BA02–C09BA15, C09DA01–C09DA09, C02AB01–C02AC05, C02DB01–C02DB04, C03DB01–C03DB02, C07AA01–C07AA06, C07AG01, C08CA01–C08DB01, C09DB01–C09DB08, C09DX01–C09DX03, C09BB02–C09BB12, C07AB03, C07AB02_1, C02KX01–C02KX05, PBS item code 9547L, 9605M
Pain	N02AA01–N02AX99, R05DA04
angina	C01DA02–C01DA70, C01DX16, C08EX02
Malignancy	L01AA01–L01AX04, L01BA01_2, L01BA03–L01XX53, L02BG03, L02BG04, L02BG06, L02BB01–L02BB04, L02BX01–L02BX03, L04AX02, L04AX04, L04AX06, L02BA01_01, L02AE03_1, L02AE02_1 **
Hyperthyroidism	'H03BA02', 'H03BB01'
Osteoporosis/Paget's	M05BA01–M05BB08, M05BX03, M05BX04, H05AA02
inflammation	M01AB01–M01AH06
Parkinson's disease	N04AA01–N04BX03
Psychotic illness	N05AA01–N05AB02, N05AB06–N05AL07, N05AX01–N05AX17
Reactive airway disease	R03AC02–R03DC03, R03DX05
Steroids-responsive conditions	H02AB01–H02AB17

Note: CHF, congestive heart failure; IHD, HTN, hypertension; **Where the ATC code has _01 or_02 at the end, the ATC code could be used for multiple indications and therefore to separate indication based on PBS item codes these additional digits were used.

Table S2. Results of poisson regression models examining the likelihood of an older adult being dispensed medication for individual chronic conditions for 2014-2016 compared to 2013*.

	**RR (95% CI), P-values**		
	**2014**	**2015**	**2016**
Diabetes	1.01 (0.99–1.02), 0.065	1.02 (1.01–1.04), < 0.001	1.03 (1.02–1.05), < 0.001
GORD	1.01 (1.00–1.02), 0.027	1.01 (1.00–1.02), 0.025	1.01 (1.00–1.02), 0.023
Hypertension	1.00 (0.99–1.00), 0.160	0.99 (0.98–0.99), 0.002	0.99 (0.98–0.99), < 0.001

Glaucoma	0.99 (0.97–1.01), 0.250	0.98 (0.96–0.99), 0.017	0.96 (0.94–0.97), < 0.001
CHF	0.99 (0.98–1.01), 0.301	0.97 (0.96–0.98), < 0.001	0.97 (0.95–0.98), < 0.001
Dementia	1.02 (0.98–1.06), 0.340	1.03 (0.98–1.07), 0.234	1.05 (1.01–1.09), 0.025
Malignancy	1.03 (1.00–1.07), 0.036	1.09 (1.06–1.13), < 0.001	1.12 (1.08–1.15), < 0.001
Osteoporosis	0.99 (0.97–1.00), 0.142	1.01 (0.99–1.02), 0.313	1.04 (1.02–1.05), < 0.001
Angina	0.94 (0.93–0.96), <0.001	0.89 (0.88–0.91), < 0.001	0.86 (0.84–0.87), < 0.001
Arrythmias	0.99 (0.97–1.01), 0.441	1.03 (1.01–1.05), 0.004	1.08 (1.06–1.10), < 0.001
Dyslipidaemia	0.98 (0.98–0.99), 0.002	0.98 (0.98–0.99), < 0.001	0.98 (0.98–0.99), < 0.001
Psychotic illness	0.98 (0.96–1.01), 0.140	0.96 (0.94–0.99), 0.005	0.95 (0.93–0.97), < 0.001
Pain	1.03 (1.02–1.04), < 0.001	1.04 (1.03–1.05), < 0.001	1.03 (1.02–1.04), < 0.001
Steroid responsive conditions	1.03, (1.02–1.05), < 0.001	1.07 (1.05–1.08), < 0.001	1.08 (1.07–1.10), < 0.001
Gout	1.00 (0.98–1.02), 0.894	1.01 (0.99–1.03), 0.170	1.01 (0.99–1.03), 0.171
Hyperthyroidism	1.03 (0.96–1.01), 0.388	1.01 (0.94–1.08), 0.769	0.99 (0.93–1.06), 0.769
Parkinson's disease	1.02 (0.99–1.06), 0.154	1.02 (0.99–1.06), 0.151	1.03 (1.00–1.07), 0.042
Epilepsy	0.97 (0.95–1.00), 0.052	0.96 (0.93–0.98), 0.003	0.95 (0.93–0.98), 0.001
Reactive airway disease	1.02 (1.00–1.03), 0.034	1.02 (1.00–1.03), 0.012	1.01 (0.99–1.023), 0.308
Inflammation/pain	0.99 (0.98–0.99), 0.019	0.97 (0.96–0.99), < 0.001	0.97 (0.96–0.98), < 0.001
Anxiety	0.97 (0.95–0.99), < 0.001	0.92 (0.91–0.94), < 0.001	0.91 (0.89–0.92), < 0.001
Depression	1.00 (0.99–1.01), 0.469	1.01 (1.00–1.02), 0.012	1.02 (1.01–1.03), < 0.001

Note: All analyses were adjusted for age and sex
